# Design and Simulation of a Simple-Structure and High-Performance Plasmonic Polarization Filter Based on Gold Layers Deposited on Photonic Crystal Fiber

**DOI:** 10.3390/mi16101088

**Published:** 2025-09-26

**Authors:** Nan Chen, Ming Zhao, Yuxin Zhu, Leilei Gao, Cheng Lu, Xingjian Sun, Xin Ding, Xianping Wang

**Affiliations:** 1 Key Laboratory of Functional Materials and Devices for Informatics of Anhui Higher Education Institutes, Fuyang Normal University, Fuyang 236037, China; 2 School of Electrical Engineering and Automation, Nantong University, Nantong 226019, China; 3 College of Physics and Optoelectronic Engineering, Shenzhen University, Shenzhen 518060, China

**Keywords:** photonic crystal fiber, surface plasmon resonance, finite element method, polarization filter, extinction ratio

## Abstract

The demand for high-performance photonic filters is steadily on the rise in the information age. This work proposed a simple-structure and high-extinction plasmonic polarization filter using gold-deposited photonic crystal fiber (PCF), by the mature finite element method (FEM). The numerical results indicate that once the structural parameters are reasonably ascertained, the operating center of this PCF filter can be verified to be at the 1.55 μm communication window. The 1-μm-long PCF filter possesses a maximum extinction ratio (ER) of −109.9 dB, with a broad operating bandwidth of 620 nm, ranging from 1.35 to 1.97 μm, and a low insertion loss (IL) of 0.3 dB. In addition, this device has an ease of fabrication based on the existing processing techniques. It is reasonable to believe that with its compact structure, comprehensive filtering performance, and high-feasibility, this all-fiber filtering device is likely to assume a crucial role in various fields, including laser technology, sensing, biomedicine, and nonlinear optics.

## 1. Introduction

With the rapid progress of modern technology, the ways of spreading information have become more diverse. With the arrival of the Internet and the big data era, the concept of the Internet of Things (IoTs) has emerged, posing a severe challenge to traditional electronic communication methods. In order to meet the demands of people’s daily life and work, information transmission is evolving towards higher transmission rates, larger transmission capacities, and lower bit error rates [1]. Nevertheless, traditional electronic communication methods have gradually fallen short of meeting the requirements of new-generation information technologies’ development, mainly because of the limitations of interference, high energy consumption, and susceptibility to heat [2]. As a new form of information carrier, optical signals have a higher carrier frequency compared to traditional electromagnetic waves. They also feature a broader bandwidth and greater information transmission capabilities. All-optical technology is the key to addressing security issues and meeting the requirements of massive information transmission. A large-capacity and ultra-high-speed all-optical network requires the support of high-performance optical devices [3]. Polarization filters, as one of the important components among them, play an important role in the construction of all-optical networks [4]. During the processing of optical signals, polarization filters modulate the amplitude and phase parameters of optical signals. Their main functions encompass the shaping of microwave photonic pulse signals, wavelength division multiplexing, and optical cross-connect systems, as well as the suppression or selection of specific light wavelengths [5,6].

Due to the continuous advancements in micro–nano technology, many novel optical waveguides can serve as carriers for polarization filtering, such as photonic crystal (PC), silicon-on-insulator (SOI), meta-surface, grating, and optical fiber [7,8,9,10,11]. Although the first four types of waveguides can achieve excellent polarization-filtering performance through rational design, their fabrication is intricate and costly. Moreover, the design flexibility is restricted, and excessive processing can render the waveguides extremely fragile. When integrated into the photonic network using optical fibers as the medium, additional losses will be incurred. Taking these factors into account, the advantages of fiber-based polarization filters become prominent. Especially after the invention of photonic crystal fibers (PCFs), through the flexible design of the cladding structure, it has become possible to achieve an unprecedented optical modulation [12]. Furthermore, the cladding holes of PCFs offer a platform for the integration of optical materials. When the transmitted light in a PCF meets specific excitation conditions, it can trigger the interaction between light and matter, thus achieving extraordinary optical effects. This enables PCFs to experience further enhancements in optical properties.

Currently, the integration of surface plasmon with PCFs for the development of new photonic devices with high performance and small size has emerged as a highly popular research area [13,14]. In a polarization filter, it is essential to create a substantial energy difference in different polarization directions to achieve stable and precise filtering performance. When noble metal materials are integrated into a PCF, as the incident light within the fiber traverses the interface between the medium and the metal, special evanescent waves are generated at this interface, commonly known as surface plasmon waves (SPWs). Once the wave vector component of the incident light along the interface between the medium and the metal coincides with the propagation constant of the surface plasmon polariton (SPP), the surface plasmon resonance (SPR) effect is excited, accompanied by a substantial release of energy [15,16]. Generally, different polarizations of light give rise to different conditions for the SPR phenomenon. By adjusting different structural parameters, the filtering performance under specific spectral windows can be achieved.

Recently, many typical PCF polarization filters have been reported. In 2018, Liu et al. designed a tunable broadband PCF SPR polarization filter using a numerical tool, and it exhibits a bandwidth of 1200 nm, covering the wavelengths from 1.0 to 2.2 μm with a high extinction ratio (ER) of −1396 dB at 1.55 μm [17]. In 2019, Hossen et al. proposed an all-fiber polarization filter based on gold nanowire-filled PCF operating around 1.42 μm; this 1 nm-long device shows the maximum ER of 253 dB with an operating bandwidth of 830 nm [18]. In 2020, Yan et al. designed a novel gold layer-deposited PCF polarization filter; the 300 μm-long polarizer possesses the maximum crosstalk (CT) of 314 dB at 1.31 μm, with a long bandwidth of 570 nm [19]. In 2021, Jiang et al. reported two ultra-broadband PCF polarization filter configurations, and the simulation results show that Structure 1 and Structure 2 with the fixed PCF length of 300 μm can achieve the CTs of −590.09 and −506.07 dB at 1.55 μm, together with the bandwidths of 1500 and 900 nm, respectively [20]. In 2022, Wang et al. put forward a multifunctional PCF deposited with gold, which has both filtering and sensing functions. When the analytes’ refractive index is 1.33, a great polarization signal difference appears at 1.52 μm. The starting wavelength of the effective filtering range is changed from 1.87 to 1.45 μm, and the bandwidth of the 1 mm-long fiber also increases from 630 to 1050 nm [21]. In 2023, Zhang et al. proposed a dual-cladding PCF SPR polarization filter, and, with the device length of only 300 μm, their filter achieves the maximum CT of 246.62 dB, with an 830 nm-long bandwidth [22]. In 2024, Ullah et al. also presented a multifunctional PCF deposited with gold films; the 2 mm-long PCF demonstrates the maximum ER of −321.54 dB at 700 nm [23]. Additionally, hollow-core fiber-based polarization filters have also been reported. The key advantages of hollow-core fiber in communication networks are ultra-low attenuation and ultra-low delay, and yet, when examining its performance as a carrier for photonic filters, the integration level and performance are somewhat lower compared to those of solid fiber-based polarization filters. In terms of engineering properties such as output, cost, and splicing, hollow-core fiber remains considerably inferior to solid PCF. Thus, there are still some challenges in the research of hollow-core fiber polarization filters [24,25].

From the above reports, it can be observed that designing a new high-performance all-fiber polarization filter by integrating the advantages of PCFs and the SPR technique holds significant research value. With the progress of semiconductor technology, materials science, optoelectronics, and other related fields, SPR technology is continuously advancing in the directions of higher selectivity, miniaturization, and intelligence. In light of the current research on diverse micro–nano structures, nanomaterials, and micro-processing techniques, fiber SPR devices, featuring different optical fiber materials or fiber micro-structures and boasting excellent sensing performance, are emerging in an endless stream. Thus, in this work, using the highly optically flexible PCF platform and complementing it with metal materials to induce the SPR effect, a new all-fiber polarization filter is reported by a numerical finite element tool [26], which features a compact size, a high ER, and a wide bandwidth within the communication band. Finally, the manufacturing process for this polarizer is also discussed.

## 2. Modeling and Theory

**Figure 1:**
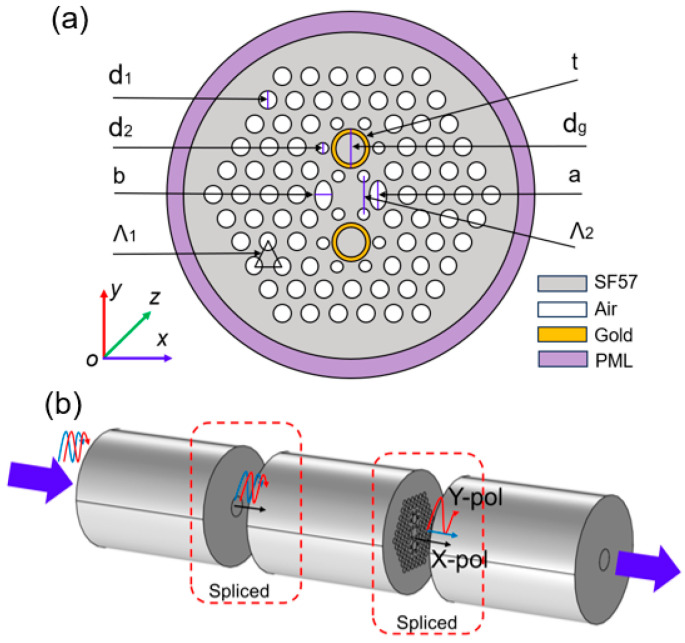
The proposed PCF SPR polarization filter scheme. (**a**) The structure of the proposed PCF SPR filter; (**b**) The schematic of the filtering process.

Figure 1a indicates the structure of the proposed PCF SPR filter. Set it so that the light beam is incident perpendicular to the x-o-y plane. The cladding structure for the proposed PCF is primarily characterized by a hexagonal shape. The diameter of the cladding air hole is denoted by *d*_1_, the diameter of the small hole is denoted by *d*_2_, and the diameter of the gold-deposited holes is denoted by *d_g_*. A pair of elliptical holes is placed on both sides of the core region, with the major axis being *a* and the minor axis being *b*. The lattice pitch (hole-to-hole spacing) is denoted by *Λ*_1_. To achieve local adjustment of the upper and lower parts of the core, the upper and lower air holes are moved closer in the direction of the core, and the distance between the upper and lower air holes is set to *Λ*_2_. The thickness of the gold layer is denoted by *t*. In addition, a cylindrical perfectly matched layer (PML), having a thickness of 1 µm, is configured around the proposed PCF structure, primarily to absorb the scattering losses at the boundary and thereby improve the accuracy of the calculation [27]. The cylindrical PML is centered at the coordinate (0, 0). In the commercial software COMSOL 5.5 Multiphysics, set perfect electric conductor (PEC) and perfect magnetic conductor (PMC) boundaries around the PCF structure. Then, the finite element operator discretizes the proposed PCF geometry into 376 vertex elements, 1880 boundary elements, 16,680 discrete triangular elements, and the maximum element size is 2.0 µm, and the minimum element size is 0.04 µm, making the number of degrees of freedom resolved by the eigenvalue solver 116,857, as shown in Figure S1 in the supplementary document. The mode analysis tool is employed to calculate the effective refractive index and mode profile of this PCF. During the finite element solution process, the variation in the error values from 1 to 10^−22^ in the iterative convergence curve indicates that the model has extremely high accuracy, which can be found in Figure S2 in the supplementary document.

This work selects SF57 glass as the background material for the proposed PCF. SF57 glass typically contains a significant amount of lead or other heavy metal oxides. These oxides are utilized to enhance the refractive index and dispersion. As a result, its properties of high refractive index, high dispersion, and nonlinearity are highly suitable for controlling the photonic bandgap. This makes SF57 glass play a crucial role in the design of precision optical systems and specialized photonic components. And the Sellmeier model of the SF57 glass is described by [28]:

(1) $$n_{SF57}(\lambda )=\sqrt{A_{0}+A_{1}\lambda^{2}+\frac{A_{2}}{\lambda^{2}}+\frac{A_{3}}{\lambda^{4}}+\frac{A_{4}}{\lambda^{6}}+\frac{A_{5}}{\lambda^{8}}}$$

where *λ* denotes the operating wavelength in μm. The detailed parameters in models are *A*_0_ = 3.24748, *A*_1_ = −0.00954782 μm^−2^, *A*_2_ = 0.0493626 μm^2^, *A*_3_ = 0.00294294 μm^4^, *A*_4_ = −1.48144 × 10^−4^ μm^6^, and *A*_5_ = 2.78427 × 10^−5^ μm^8^, respectively.

Based on the studies in the previous section, it is evident that gold is the most frequently used material for SPR excitation in PCF filters. The main reason is that, while the SPR excitation efficiency of gold is not optimal, silver and aluminum are highly susceptible to oxidation. When utilized in the design of photonic devices, these metal materials necessitate additional material protection measures. Consequently, gold is selected as the SPR excitation material in this filter. The gold permittivity can be described by the Drude–Lorentz model [29], and it is as follows:

(2) $$\varepsilon_{r}(\omega )=1-\frac{\Omega_{p}^{2}}{\omega \cdot (\omega -i\Gamma_{0})}+\sum_{j=1}^{m}\frac{f_{j}\cdot \omega_{p}^{2}}{(\omega_{j}^{2}-\omega^{2})+i\omega \cdot \Gamma_{j}}$$

where Ω*_P_* denotes the plasma frequency related to the oscillator strength ƒ_0_ and damping constant Γ_0_; *ω_P_* denotes the plasma frequency; and *m* denotes the number of oscillators related to frequency *ω_j_*, strength *ƒ_j_*, and lifetime 1/Γ*_j_*. The detailed parameters for the gold model are shown in Table 1 [30,31].

Typically, PCF filters need to be spliced to single-mode fibers (SMFs) at both ends to create a sandwich structure, as depicted in Figure 1b. When the mixed polarized light passes through the left SMF and enters the PCF filter, differences in the SPR effect cause a significant variation in signal intensity at the set wavelength. As a result, the desired polarization signal is output at the right output end. Here, the confinement loss (CL) is utilized to describe the x-pol and y-pol signals, which can be expressed by the following [32]:

(3) CL(dB/mm)=8.686×2πλ×Im(neff)×103

where Im(*n_eff_*) represents the imaginary part of the effective refractive index (ERI) for polarized modes.

The normalized output power (NOP) of different polarization modes in this filter is evaluated by the following equation [33]:

(4) $$P_{out}(x,y)=P_{in}(x,y)\times \exp(-CL(x,y)\times L\times \frac{Ln(10)}{10})$$

where *L* represents the PCF’s length, and *P_in_*(*x*, *y*) and *P_out_*(*x*, *y*) represent normalized input and output power, respectively. Here, assuming *P_in_*(*x*, *y*) = 1.

In order to analyze the filtering performance, the extinction ratio (ER) is employed to evaluate the characteristics of the proposed PCF filter, and it can be described by the following [34]:

(5) $$ER(\mathrm{dB})=10\cdot \log_{10}(\frac{P_{out}(x)}{P_{out}(y)})$$

where *P_out_*(*x*) and *P_out_*(*y*) represent output power of x- and y-pol direction, respectively.

In practical engineering, insertion loss (IL) involves a variety of factors, such as splicing loss, transmission loss, and mode conversion loss, etc. In this work, IL is estimated by the core mode transmission loss and is employed to assess the reliability of the filtering process, which can be defined as follows [35]:

(6) $$IL(dB)=-10\cdot \log_{10}(\frac{P_{out}}{P_{in}})$$

where *P_in_* and *P_out_* represent input power and output power, respectively. Here, *P_in_* equals the sum of *P*(*x*) and *P*(*y*).

## 3. Results and Discussion

### 3.1. Dispersion Relationship

**Figure 2:**
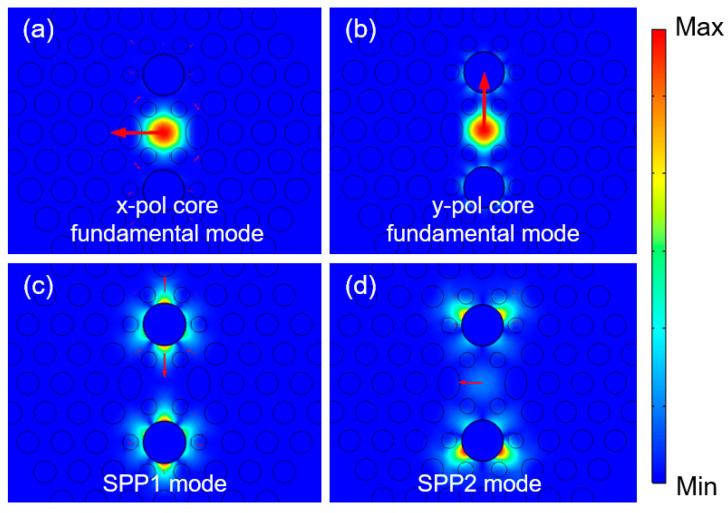
The supported mode distributions in the proposed PCF filter. (**a**) x-pol core fundamental mode; (**b**) y-pol core fundamental mode; (**c**) SPP1 mode; (**d**) SPP2 mode.

Building upon the principle of total internal reflection and the principle of photon crystal transmission, the fundamental mode fields inside the fiber can be effectively manipulated by adjusting the arrangement of air holes in the PCFs’ cladding. Moreover, after the deposition of the gold layers, additional plasmonic modes can be generated. As shown in Figure 2, there are (a) x-pol core fundamental modes, (b) y-pol core fundamental modes, (c) SPP1 modes, and (d) SPP2 modes, respectively, in the proposed PCF. According to classic coupled-mode theory [36,37], investigate the mode coupling between the fundamental core modes and the SPP modes, and acquire the energy interaction of different combinations.

**Figure 3:**
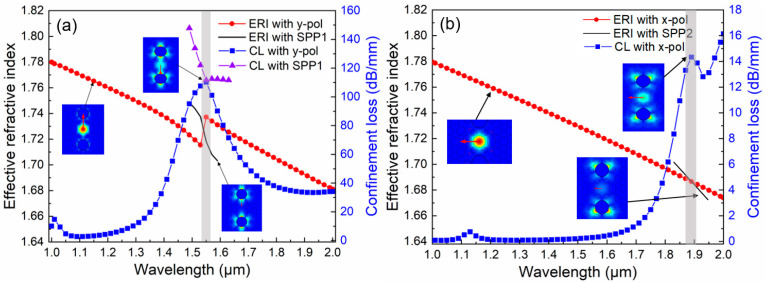
Dispersion relationship between core fundamental modes and plasmonic modes when *d*_1_ = 1.1 μm, *d*_2_ = 0.7 μm, *d_g_* = 1.9 μm, *a* = 1.5 μm, *b* = 1.0 μm, *Λ*_1_ = 1.5 μm, *Λ*_2_ = 2.04 μm, and *t* = 50 nm. (**a**) in y-pol direction; (**b**) in x-pol direction.

In optical devices incorporating the SPR effect, when the core fundamental mode couples with the plasmonic mode, the energy loss of the core fundamental mode gradually increases and attains its maximum value at the resonant wavelength. It can be obtained that because the small hole is nearest to the gold ring layer, the excited SPP mode is reshaped, and two SPR processes with significantly different energy release can be achieved from Figure 3. Figure 3a shows that the y-pol core fundamental mode and the SPP1 mode can interact with one another. They satisfy the phase-matching condition at 1.55 μm, giving rise to a distinct resonance mode with substantial energy interaction. This generates a pronounced SPR phenomenon and releases a significant amount of energy. In accordance with the ERI with SPP1 and CL with SPP1 curves, the interaction between the y-pol mode and the SPP1 mode represents a complete coupling process, and the detailed process can be found in Figure S3 in supplementary document. Consequently, the CL with the SPP1 curve demonstrates a physical avoided crossing phenomenon [38]. Figure 3b depicts that the x-pol core fundamental mode and the SPP2 mode interact at 1.88 μm. Although an SPR effect can also be produced, its intensity is notably weaker than that of the former case. Differences exist between the two resonance processes. Given that the small holes around the metal ring layer are relatively close, the two plasmonic modes are reshaped. It can be observed that a greater amount of the SPP1 mode’s energy interacts with the y-pol mode, while more energy of the SPP2 mode leaks to the upper and lower sides, thereby participating less in the overall SPR process. Specifically, the CL of the y-pol mode at 1.55 μm is 110.2 dB/mm, and that of the x-pol mode is only 0.3 dB/mm. These phenomena lay a prerequisite for the design of a high-performance in-fiber polarization filter operating at the 1.55 μm communication window.

### 3.2. The Effect of Structural Parameters on Cl

**Figure 4:**
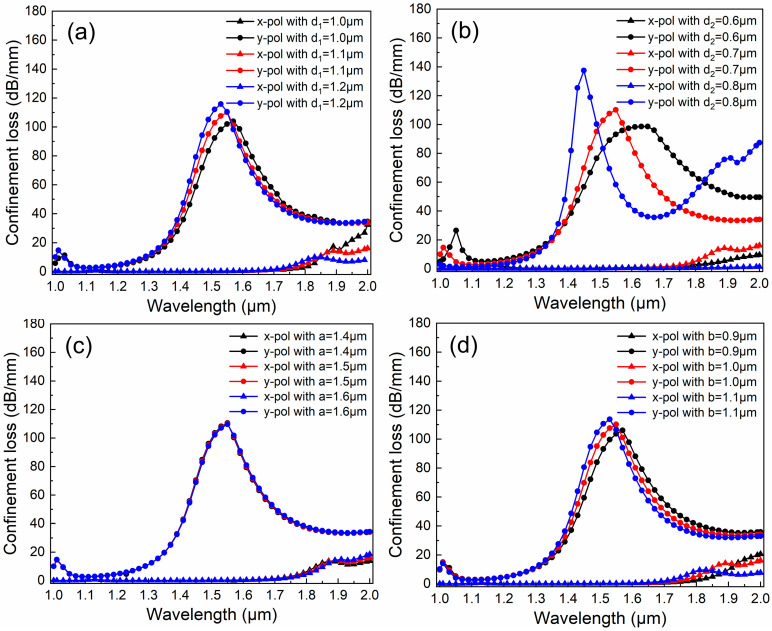
The effect of different apertures on the CL curves. (**a**) The effect of diameter *d*_1_ on CL; (**b**) The effect of diameter *d*_2_ on CL; (**c**) The effect of long axis *a* on CL; (**d**) The effect of short axis *b* on CL.

**Figure 5:**
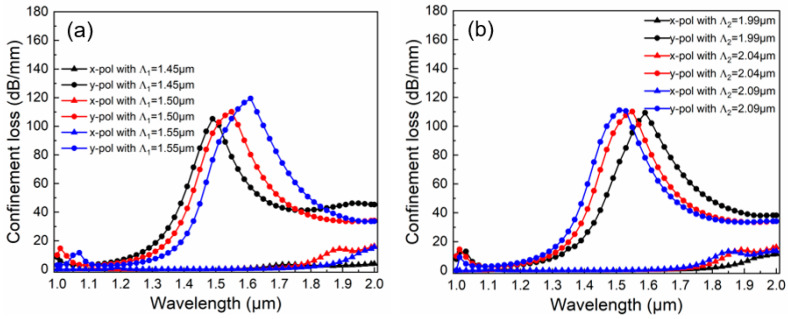
The effect of hole-to-hole space on the CL curves. (**a**) The effect of pitch *Λ*_1_ on CL; (**b**) The effect of pitch *Λ*_2_ on CL.

**Figure 6:**
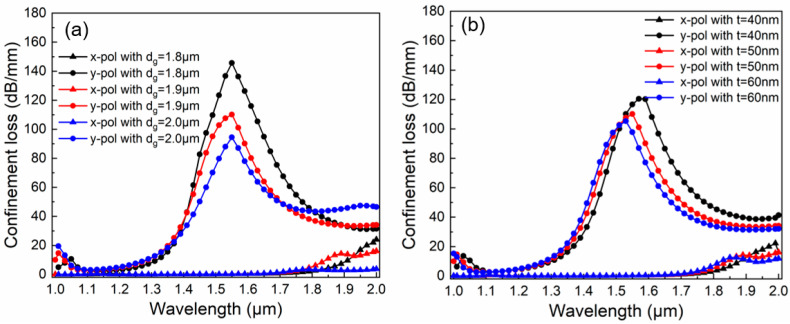
The effect of plasmonic material factors on the CL curves. (**a**) The effect of gold-deposited hole diameter *d_g_* on CL; (**b**) The effect of gold layer thickness *t* on CL.

Generally, it is challenging to guarantee the absolute accuracy of the PCFs’ structural parameters. Even after manufacturing, there will invariably be minor parameter fluctuations. Thus, the performance of the PCF filter is sensitive to structural change. In the section, the common control variate method is employed to analyze the effect of the structural parameters on CL, as shown in Figure 4, Figure 5 and Figure 6.

Figure 4a depicts the effect of diameter *d*_1_ on CL when *d*_2_ = 0.7 μm, *d_g_* = 1.9 μm, *a* = 1.5 μm, *b* = 1.0 μm, *Λ*_1_ = 1.5 μm, *Λ*_2_ = 2.04 μm, and *t* = 50 nm. The value of *d*_1_ takes from 1.0 to 1.2 μm, at intervals of 0.1 μm. The cladding holes *d*_1_ are situated relatively far from the core region, and their influence on the optical properties of the proposed PCF is somewhat limited. This is mainly manifested in the change in the overall mode effective refractive index (ERI). As *d*_1_ increases, the proportion of the related medium decreases, and the ERI also declines. Consequently, the resonance wavelength in the y-pol direction shifts towards the blue end of the spectrum, with corresponding wavelengths of 1.57 μm, 1.55 μm, and 1.53 μm, respectively. Simultaneously, as the proportion of the core region becomes larger and larger, more energy is confined within the core and resonates with the SPP1 mode. As a result, the energy increases, albeit relatively moderately. The corresponding CL values are 103.9 dB/mm, 110.2 dB/mm, and 115.8 dB/mm, respectively.

Figure 4b depicts the effect of diameter *d*_2_ on CL when *d*_1_ = 1.1 μm, *d_g_* = 1.9 μm, *a* = 1.5 μm, *b* = 1.0 μm, *Λ*_1_ = 1.5 μm, *Λ*_2_ = 2.04 μm, and *t* = 50 nm. The value of *d*_2_ is varied from 0.6 to 0.8 μm at intervals of 0.1 μm. The holes *d*_2_ are positioned around the gold-deposited hole and also exert a certain influence on the core. On one hand, it can reshape the SPP mode; on the other hand, it will also bring about changes in the core area. As *d*_2_ increases, the core region shrinks, and the ERI of the y-pol mode decreases. Consequently, the resonance peak shifts towards the blue region of the spectrum. The corresponding wavelengths are 1.63 µm, 1.55 µm, and 1.45 µm, respectively. Owing to the holes squeezing the SPP1 mode, a greater amount of SPP energy participates in the resonance process. As a result, the resonance intensity gradually rises. The corresponding CL intensities are 98.6 dB/mm, 110.2 dB/mm, and 137.4 dB/mm, respectively.

Figure 4c depicts the effect of long axis *a* on CL when *d*_1_ = 1.1 μm, *d*_2_ = 0.7 μm, *d_g_* = 1.9 μm, *b* = 1.0 μm, *Λ*_1_ = 1.5 μm, *Λ*_2_ = 2.04 μm, and *t* = 50 nm. The value of *a* is varied from 1.4 to 1.6 μm at intervals of 0.1 μm. The elliptical hole modulation is introduced on both sides of the core. Variation in the major axis has a minimal impact on this PCF. The CL curves basically overlap. The resonance wavelengths remain unchanged, with the corresponding resonant intensities being 110.6 dB/mm, 110.2 dB/mm, and 109.7 dB/mm, respectively. Overall, this influence can be neglected.

Figure 4d depicts the effect of short axis *b* on CL when *d*_1_ = 1.1 μm, *d*_2_ = 0.7 μm, *d_g_* = 1.9 μm, *a* = 1.5 μm, *Λ*_1_ = 1.5 μm, *Λ*_2_ = 2.04 μm, and *t* = 50 nm. The value of *a* is varied from 0.9 to 1.1 μm at intervals of 0.1 μm. Distinct from the role of the long axis, the variation in the short axis *b* directly influences the size of the core. The CL curve of this PCF will vary in accordance with the change in *b*. As *b* increases, the core area diminishes. The ERI curve shows an overall decline, and the resonance point shifts towards the short wavelength. The corresponding wavelengths are 1.57 µm, 1.55 µm, and 1.53 µm, respectively. The increase in *b* squeezes the core, enabling more energy to participate in the SPR process. Consequently, the resonance intensity gradually rises. The corresponding CL values are 106.0 dB/mm, 110.2 dB/mm, and 94.0 dB/mm, respectively.

PCFs are constructed by applying the principle of photonic crystal transmission to optical fiber waveguides. Thus, the lattice constant, which represents the hole-to-hole space within the PCF, dictates its transmission mechanism. The influence of a minor change in the spacing proves to be more substantial than that resulting from a change in the aperture. Figure 5a shows the effect of pitch *Λ*_1_ on CL when *d*_1_ = 1.1 μm, *d*_2_ = 0.7 μm, *d_g_* = 1.9 μm, *a* = 1.5 μm, *b* = 1.0 μm, *Λ*_2_ = 2.04 μm, and *t* = 50 nm. The value of *Λ*_1_ takes from 1.45 to 1.55 μm at intervals of 0.05 μm. As *Λ*_1_ increases, the density of the background medium increases, and the ERI shows an overall upward trend. Consequently, the resonance wavelength in the y-pol direction produces a redshift. The corresponding wavelengths are 1.49 µm, 1.55 µm, and 1.61 µm, respectively. Since more medium restricts the energy transmission within the core region, more energy coupling takes place, and thus the CL intensity increases accordingly. The corresponding CL values are 105.2 dB/mm, 110.2 dB/mm, and 119.4 dB/mm, respectively. In this PCF, the spacing between the upper and lower holes near the core region is changed in order to achieve the adjustment of the resonance point without any alteration in the CL strength. Figure 5b shows the effect of pitch *Λ*_2_ on CL when *d*_1_ = 1.1 μm, *d*_2_ = 0.7 μm, *d_g_* = 1.9 μm, *a* = 1.5 μm, *b* = 1.0 μm, *Λ*_1_ = 1.5 μm, and *t* = 50 nm. The value of *Λ*_2_ takes from 1.99 to 2.09 μm at intervals of 0.05 μm. It can be observed that as *Λ*_2_ increases, the resonance peak produces a blueshift. The corresponding wavelengths are 1.59 µm, 1.55 µm, and 1.51 µm, respectively. This is primarily because *Λ*_2_ is utilized to adjust the shape of the core. A slight increase in *Λ*_2_ will lead to a decrease in the ERI, while the area of the mode field remains relatively stable. As a result, the energy change is almost negligible. Hence, by leveraging this property of *Λ*_2_, an accurate filtering central wavelength can be achieved by adjusting the value of *Λ*_2_.

Finally, the effect of plasmonic material factors on the CL curves is discussed. Figure 6a displays the effect of gold-deposited hole diameter *d_g_* on CL when *d*_1_ = 1.1 μm, *d*_2_ = 0.7 μm, *a* = 1.5 μm, *b* = 1.0 μm, *Λ*_1_ = 1.5 μm, *Λ*_2_ = 2.04 μm, and *t* = 50 nm. The value of *d_g_* takes from 1.8 to 2.0 μm at intervals of 0.1 μm. It is readily observable that the primary function of *d_g_* is to modulate the resonance energy in the y-pol direction. As the value of *d_g_* rises, the energy weakens. This is because an increase in *d_g_* further compresses the SPP1 mode. Consequently, less SPP energy is exchanged with the core fundamental mode. Leveraging this phenomenon, appropriately decreasing *d_g_* is conducive to enhancing the filtering ability. Figure 6b displays the effect of gold layer thickness *t* on CL when *d*_1_ = 1.1 μm, *d*_2_ = 0.7 μm, *d_g_* = 1.9 μm, *a* = 1.5 μm, *b* = 1.0 μm, *Λ*_1_ = 1.5 μm, and *Λ*_2_ = 2.04 μm. The values of *t* are 40 nm, 50 nm, and 60 nm, respectively. As *t* increases, the resonance wavelength experiences a blue shift. The corresponding wavelengths are 1.57 μm, 1.55 μm, and 1.53 μm, respectively. Meanwhile, the resonance energy decreases, with the corresponding resonance energy values being 120.4 dB/mm, 110.2 dB/mm, and 105.3 dB/mm, respectively. Thus, the proper thickness of the gold layer can influence both the resonance point and the filtering ability simultaneously. Evidently, a thickness of 50 nm is more suitable for filtering applications in the 1.55 μm communication window.

### 3.3. Filtering Performance Analysis

**Figure 7:**
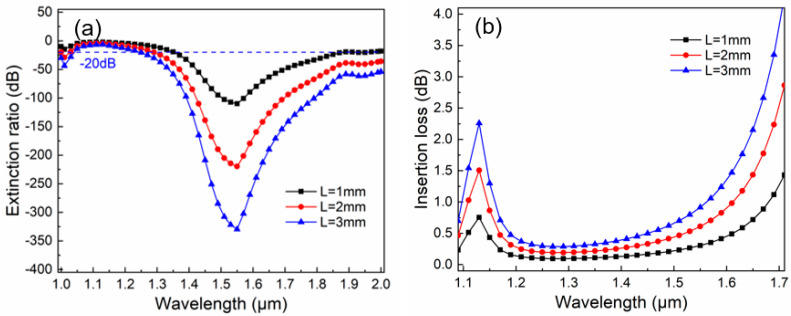
The filtering performance in the proposed PCF filter with different device lengths when *d*_1_ = 1.1 μm, *d*_2_ = 0.7 μm, *d_g_* = 1.9 μm, *a* = 1.5 μm, *b* = 1.0 μm, *Λ*_1_ = 1.5 μm, *Λ*_2_ = 2.04 μm, and *t* = 50 nm. (**a**) The ER curves versus different lengths; (**b**) The IL curves versus different lengths.

According to the analysis in the preceding paragraph, the structural parameters are set as *d*_1_ = 1.1 μm, *d*_2_ = 0.7 μm, *d_g_* = 1.9 μm, *a* = 1.5 μm, *b* = 1.0 μm, *Λ*_1_ = 1.5 μm, *Λ*_2_ = 2.04 μm, and *t* = 50 nm. This gold-coated PCF can serve as the central operating component of an optical filter operating at 1.55 μm. A detailed exploration of its filtering performance is displayed in Figure 7.

Figure 7a depicts the ER curves versus different lengths in the proposed PCF filter. The length of the proposed PCF filter has a direct impact on its filtering capability and operating bandwidth (BW). The blue dotted line represents the ER = −20 dB boundary line. This line is conducive to determining the range of the BW. When this PCF’s length is 1 mm, its maximum ER is −109.9 dB at 1.55 μm, with the BW of 640 nm, spanning from 1.35 to 1.97 μm, showing a high filtering property and multi-wavelength selectivity. If it is necessary to support both the 1.31 μm and 1.55 μm communication windows, the integration of this filter can be moderately compromised. When *L* = 2 mm, the central ER can reach −219.8 dB. Starting from a wavelength of 1.28 μm, the ER of this filter is better than 20 dB throughout. In the investigated wavelength band, the BW can cover 720 nm. If the device length is further increased, when *L* = 3 mm, the ER at 1.55 μm reaches as high as −330.0 dB, and its BW is further broadened. Considering both the integration level of the device and the practical performance, it is advisable to set the length of the proposed PCF to 1 mm.

Figure 7b shows the IL curves versus different lengths in the proposed PCF filter. IL directly influences the transmission efficiency and quality of optical communication systems. In a filter, IL is determined by multiple factors, including material absorption and scattering, manufacturing process precision, operating wavelength, and BW, etc. In this work, we focus only on the fundamental properties of the material to analyze IL in this PCF. It can be seen that as *L* increases, the IL curve generally shows an upward trend. This indicates that the length of this device is directly proportional to the increment in IL. When the length is shorter, the IL performance is more favorable. Evidently, when *L* = 1 mm, within the range of 1.3–1.6 μm near the operating center, the IL remains below 0.5 dB. Specifically, the IL at 1.55 μm is 0.3 dB. This suggests that this filter exhibits good filtering stability.

Summarizing the previous content, the filtering performance changes resulting from the fluctuation of structural parameters are presented in Table 2. It can be obtained that, based on the variations in filtering performance induced by the fluctuations of the investigated structural parameters, regarding the central operating wavelength, the maximum variation is within 0.1 μm. The maximum change in the ER value is 35.5 dB, the maximum bandwidth change reaches 140 nm, and the change in IL remains within 0.18 dB. Given that the operating central wavelength of this PCF filter is at 1.55 μm, with an ER of −109.9 dB, an operating bandwidth of 620 nm, and a low IL at 1.55 μm of 0.3 dB, it still maintains stable filtering characteristics in the face of changes in structural parameters, indicating good tolerance compatibility.

In addition, the performance comparison between this filter and the reported PCF filters is performed, as depicted in Table 3. Currently, most PCF filters primarily operate within common communication windows. Their lengths are on the order of millimeters, and there is a trend towards further reduction. In terms of extinction capability, the maximum ER can reach up to 4520 dB. However, an ER in the hundreds of decibels is generally sufficient to achieve a satisfactory filtering effect. Regarding the BW, a wider bandwidth implies more wavelength options, which is advantageous for expanding new transmission bands under the current limited communication channel resources. Concerning the IL, this PCF filter exhibits the best IL value at the central wavelength among the surveyed literature.

In recent years, the proposed PCF filter, along with those in references [46,47], has been based on the conventional hexagonal lattice structure. In these schemes, the holes around the core are finely adjusted, and the gold film is utilized to stimulate the SPR effect for realizing filtering performance. The hexagonal lattice structure is stable and can be easily fabricated in the existing manufacturing process. Compared with the all-fiber filter in [46], which has a relatively large length, and the design in [47], where IL was not considered, and also in contrast to the PCF filter designs of other scholars, although the performance of the proposed PCF filter may not be superior in every aspect, when multiple factors are considered, it demonstrates good overall performance. This simple-structure and high-performance in-fiber polarizer is anticipated to make contributions to the construction of the all-optical communication network.

## 4. Fabrication Discussion

**Figure 8:**
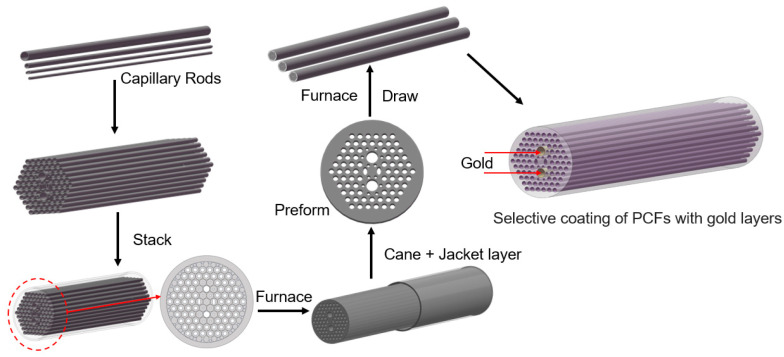
The manufacturing process for the proposed PCF filter.

It is necessary to explore the feasibility of fabricating this PCF filter by leveraging existing micro–nano processing technologies. The development of this all-fiber filter entails the manufacture of the PCF and the selective deposition of the gold layers, as shown in Figure 8.

In PCF manufacture, the mature stack and draw method can be selected to fabricate the proposed PCF [48,49]. The general process is as follows: Firstly, prepare several SF57 glass capillaries. Stack them in the form shown in Figure 1. Place a solid glass rod at the center as the core, and use SF57 glass tubes on the outside to fix the overall structure, and then draw the primary PCF cane. Next, add a jacket layer to the outside of the cane rod. Put the assembled preform into a fiber drawing tower and soften it at a high temperature. Stretch it into a primary preform with a diameter of 2–5 mm and perform a secondary stretching to obtain the PCF preform. During the drawing stage, put the preform into the fiber drawing tower again. Precisely control the temperature in a gradient manner, adjust the air pressure and the feeding speed, and, finally, draw the desired PCF. If conditions permit, a protective layer can be coated on the outside of the proposed PCF and then cured by ultraviolet light for protection.

In the selective deposition of the gold layers, a dependable chemical vapor deposition method can be utilized to deposit gold layers into the inwalls of PCF’s *d_g_* microchannels [50,51]. The general process is as follows: Prepare a piece of the proposed PCF with a length of ~ 5 cm. Firstly, for the holes, except the *d_g_* holes, they are sealed using methods like UV-curable adhesives. Next, a gaseous gold ion solution is made to react with a reducing agent inside the unblocked air holes. The deposition time is precisely regulated so as to attain the desired thickness of the gold thin film. Then, the base liquid is heated to allow evaporation, resulting in the formation of dense gold layers. Subsequently, these gold layers are cleaned by passing an inert gas through them.

Finally, the gold-coated PCF at both ends is fused with SMFs to construct a sandwich structure. The PCF-SMF splice loss can be estimated by utilizing the mode-field diameters of the proposed PCF and an SMF. The mode-field diameter of this PCF is ~2.34 μm at 1.55 μm. Assuming that lateral offset and axial tilt are neglected, the splice loss produced when this PCF is spliced with an SMF having a mode-field diameter of 10 μm is ~7.06 dB [52]. The calculation process can be found in the supplementary document. The reduction in the splice loss can be effectively regulated through the splice-free interfacing or the repeated arc discharge method [53]. Subsequently, these components are connected in sequence following the order of the light source, SMF-PCF-SMF, and the optical spectrum analyzer. The actual performance of the proposed PCF filter is assessed by analyzing the light signals’ information detected in the spectrometer [54,55].

## 5. Conclusions

In conclusion, this work demonstrates a simple-structure and high-performance in-fiber plasmonic filter based on gold layers deposited on PCF, using the finite element simulation tool. Numerical simulation shows that, by making use of the flexible adjustment ability of the PCF platform and the SPR effect of the gold layer, when the structural parameters including *d*_1_ = 1.1 μm, *d*_2_ = 0.7 μm, *d_g_* = 1.9 μm, *a* = 1.5 μm, *b* = 1.0 μm, *Λ*_1_ = 1.5 μm, *Λ*_2_ = 2.04 μm, and *t* = 50 nm are determined, the CL of the y-pol mode at 1.55 μm is 110.2 dB/mm, and that of the x-pol mode is only 0.3 dB/mm, an all-fiber filter operating in the 1.55-μm communication window can be constructed. This 1 mm-long PCF filter exhibits a maximum ER at 1.55 μm of −109.9 dB, a broad BW of 620 nm, ranging from 1.35 to 1.97 μm, and a low IL at 1.55 μm of 0.3 dB. What is more, the manufacturing scheme is also investigated, showing a relatively high feasibility. Featuring small size and excellent filtering performance, the proposed PCF filter offers a candidate device for all-optical networks characterized by low energy consumption, high bandwidth, and low latency. This significantly contributes to the integration and intelligent development of optical communication networks in the future.

## Figures and Tables

**Table 1:** The detailed parameters of gold wire in Drude–Lorentz model.

Parameter	*ω_p_*	*m*	*f*_0_	Γ_0_	*ƒ*_1_	Γ_1_	*ω*_1_	*ƒ*_2_	Γ_2_	*ω*_2_
Value	12.84	5	0.760	0.053	0.024	0.241	0.415	0.010	0.345	0.830
Parameter	*ƒ*_3_	Γ_3_	*ω*_3_	*ƒ*_4_	Γ_4_	*ω*_4_	*ƒ*_5_	Γ_5_	*ω*_5_	
Value	0.071	0.870	2.969	0.601	2.494	4.304	4.384	2.214	13.32	

**Table 2:** Performance comparison between this filter with different structural parameters.

Strut. Para.	Δ*λ_res_* (μm)	ΔER (dB)	ΔBW (nm)	ΔIL (dB)
*d*_1_ + 0.1 μm	+0.02	−6.4	+10	−0.02
*d*_1_ − 0.1 μm	−0.02	+5.7	−140	+0.09
*d*_2_ + 0.1 μm	+0.08	−12.3	+10	−0.06
*d*_2_ − 0.1 μm	−0.10	+27.4	+10	+0.03
*d_g_* + 0.1 μm	0	+35.5	0	+0.18
*d_g_* − 0.1 μm	0	−15.7	−100	−0.2
*a* + 0.1 μm	0	+0.3	0	0
*a* − 0.1 μm	0	−0.6	0	0
*b* + 0.1 μm	+0.02	−4.2	−70	−0.18
*b −* 0.1 μm	−0.02	+3.4	+10	+0.16
*Λ*_1_ + 0.05 μm	−0.06	−4.9	−50	+0.16
*Λ*_1_ − 0.05 μm	+0.06	+9.1	+40	−0.1
*Λ*_2_ + 0.05 μm	+0.04	−1.0	+30	+0.14
*Λ*_2_ − 0.05 μm	−0.04	+0.9	−30	−0.17
*t* + 10 nm	+0.02	+10.2	−130	+0.14
*t −* 10 nm	−0.02	−4.9	−40	−0.19

**Table 3:** Performance comparison between this filter and the reported PCF filters.

Ref.	Res. Wave. (µm)	Length (mm)	Max. ER (dB)	BW (nm)	IL (dB)
[39]	1.887	1	/	1552	/
[40]	1.55	2	4520	/	/
[41]	1.56	1	133	>800	0.59
[42]	1.06	0.1	>25	400	/
[43]	1.31/1.55	0.5	588.2/370.6	640	/
[44]	1.31	0.4	249.1	>880	/
[45]	1.55	6	734	390	/
[46]	1.25	2	478	750	~0.48
This work	1.55	1	109.9	620	0.3

## Data Availability

This paper is a theoretical work, and the data, graphs, and programs generated for this work can be made available on request from the corresponding author.

## Supplementary Materials

The following supporting information can be downloaded at: https://www.mdpi.com/article/10.3390/mi16101088/s1, Figure S1: The triangulation mesh of the proposed PCF filter; Figure S2. Iterative convergence curves during parametric sweeping in the simulation; Figure S3. The physical avoided crossing between the y-pol mode and the SPP1 mode (complete coupling).
