# Supplementary material for: Design and Simulation of a Simple-Structure and High-Performance Plasmonic Polarization Filter Based on Gold Layers Deposited on Photonic Crystal Fiber
Source: Micromachines (Basel). 2025 Sep 26;16(10):1088. doi: 10.3390/mi16101088 (PMC12565939; doi:10.3390/mi16101088)
Supplement: Supplementary file 1 [file micromachines-16-01088-s001.zip › micromachines-3867120-supplementary.pdf]

Supplementary document

1. *Geometric structure meshing*

In the commercial software COMSOL Multiphysics, set perfect electric conductor (PEC) and perfect magnetic conductor (PMC) boundaries around the PCF structure. Then, the finite element operator discretizes the proposed PCF geometry into 376 vertex elements, 1880 boundary elements, 16680 discrete triangular elements, the maximum element size is 2.0 μm and the minimum element size is 0.04 μm, making the number of degrees of freedom resolved by the eigenvalue solver 116,857, as shown in Figure S1. The mode analysis tool is employed to calculate the effective refractive index and mode profile of this PCF. Subsequently, post-processing is carried out to acquire its filtering performance.

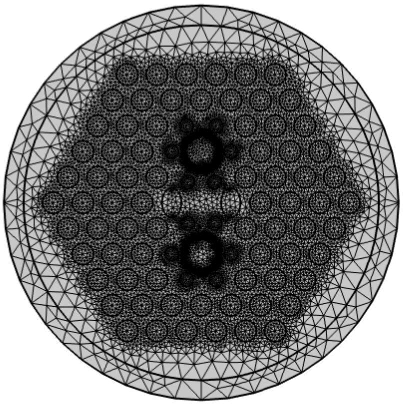

Figure S1. The triangulation mesh of the proposed PCF filter.

2. *Convergence curves*

Convergence analysis can assess the accuracy of numerical calculations through parametric sweeps. As depicted in Figure S2, at the onset of iteration, a high error value suggests that the initial prediction error of the model is substantial. As the number of iterations progresses, the error value steadily declines, indicating that the model enhances its data-fitting ability by continuously adjusting parameters. When the error value reaches an exceedingly low level, signifying that the model's prediction error is minuscule. Therefore, the reduction of the error value from 1 to ~10^{-22} approximately demonstrates that the model exhibits extremely high accuracy.

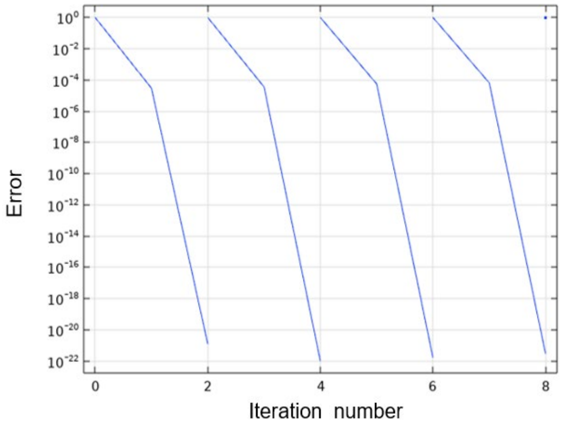

Figure S2. Iterative convergence curves during parametric sweeping in the simulation.

3. *The complete coupling process between y-pol mode and SPP1 mode*

We have calculated the data around 1.55 μm and the mode field distributions, as presented in Figure S3. Figure S3 shows that in accordance with CL with SPP1 curves, the interaction between the y-pol mode and the SPP1 mode represents a complete coupling process. Consequently, the CL with SPP1 curve demonstrates a physical avoided crossing phenomenon.

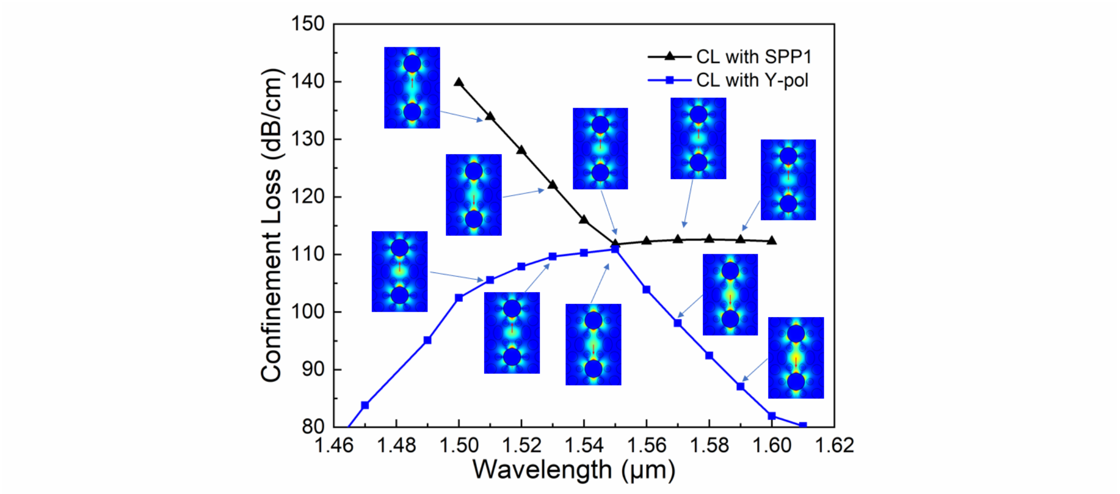

Figure S3. The physical avoided crossing between y-pol mode and SPP1 mode (complete coupling).

4. *Calculation of PCF-SMF splice loss*

If we ignore the lateral deviation and axial tilt of the optical fiber during timing alignment, the splice loss can be defined as [1]

$$L_s = 20 \cdot \log[\frac{1}{2} \cdot (\frac{MFD_{PCF}}{MFD_{SMF}} + \frac{MFD_{SMF}}{MFD_{PCF}})] \tag{1}$$

Where, MFD_{PCF} and MFD_{SMF} are the mode field diameters of the proposed PCF and a SMF, respectively. MFD_{PCF} can be expressed as [1]

$$MFD_{PCF} = \sqrt{\frac{1}{2} \cdot (MFD_x^2 + MFD_y^2)} \tag{2}$$

Where, MFD_x and MFD_y are calculated from the intensity field distribution along the two polarization directions based on COMSOL software. It can be calculated that the MFD_{PCF} is ~2.34 μm at 1.55 μm. Thus, when the value of MFD_{SMF} is taken as 10 μm, the PCF-SMF splice loss of ~7.06 dB can be achieved.

Reference:

[1] Bala A, Chowdhury K R, Mia M B, et al. Highly birefringent, highly negative dispersion compensating photonic crystal fiber[J]. Applied Optics, 2017, 56(25): 7256-7261.
